# *greenfeedr*: An R package for processing and reporting GreenFeed data

**DOI:** 10.3168/jdsc.2024-0662

**Published:** 2024-12-12

**Authors:** Guillermo Martinez-Boggio, Patrick Lutz, Meredith Harrison, Kent A. Weigel, Francisco Peñagaricano

**Affiliations:** 1 Department of Animal and Dairy Sciences, University of Wisconsin–Madison, Madison, WI 53706; 2 C-Lock Inc., Rapid City, SD 57703

## Abstract

Greenhouse gases produced by livestock are important contributors to climate change. The ability to measure large-scale exhaled metabolic gases from cattle using GreenFeed systems will help farmers to reduce enteric emissions while maintaining or increasing cow productivity. GreenFeed units are portable chamber systems that measure individual animal gas production in real time. Thus, the machines generate large amounts of daily data that can be overwhelming for users to process. This challenge motivated us to develop an R package named *greenfeedr* that offers functions for downloading, processing, and reporting GreenFeed data. Herein, we describe all functions implemented in the *greenfeedr* R package and present examples based on dairy cow data. The R package has functions for downloading GreenFeed data (*get_gfdata*), for generating daily and final reports (*report_gfdata*), for processing daily and final records (*process_gfdata*), and extra functions that help to extract information regarding pellet intakes and daily visits (*pellin* and *viseat*). Using our example data with 32 lactating dairy cows, we demonstrated the capabilities of the different functions to generate easy-to-read reports and process large amount of data. Also, we included in the function *process_gfdata* some parameters that will help users to define the best criteria to process their own GreenFeed data. Overall, *greenfeedr* represents an important advancement in the management and analysis of GreenFeed data, offering an efficient tool tailored to the needs of the user.

## Highlights

• *Greenfeedr* is an R package for processing GreenFeed data.
• Functions *report_gfdata* processes and reports daily and finalized GreenFeed data.
• Function *process_gfdata* processes GreenFeed data for subsequent analysis.
• Functions *pellin* and *viseat* help to process pellet intakes and GreenFeed visits.

## Graphical Abstract

**Figure:**
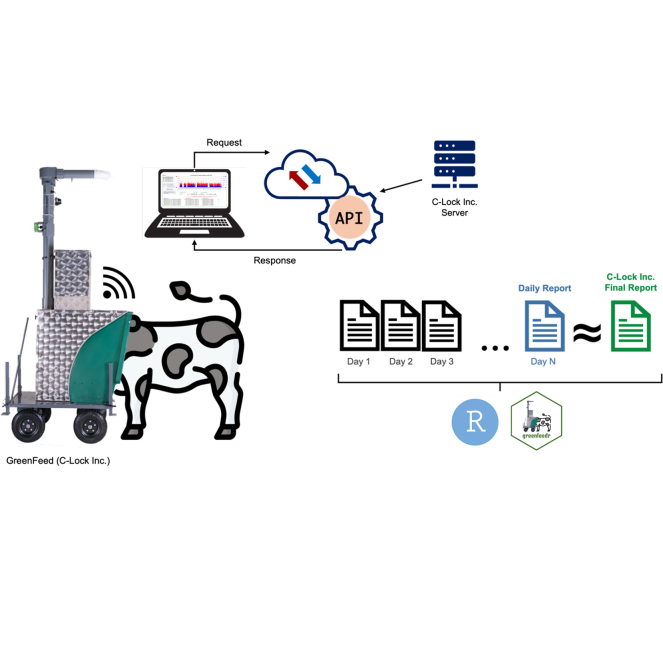
**Summary:** Greenhouse gases produced by livestock are important contributors to climate change. GreenFeed units are portable chamber systems that measure individual animal gas production in real time. This system generates large amounts of daily data that can be overwhelming for users to process. This challenge motivated the development of an R package named *greenfeedr* that contains functions for downloading, processing, and reporting GreenFeed data. This R package has a function for downloading GreenFeed data (*get_gfdata*), functions for generating daily and final reports (*report_gfdata*), functions for processing daily and final records (*process_gfdata*), and functions that help to process pellet intakes and daily visits (*pellin* and *viseat*). Overall, the *greenfeedr* represents an important advancement in the management and analysis of GreenFeed data, offering an efficient tool tailored to the needs of the user.

Climate change due to GHG produced by ruminants is a hot topic. The goal is to reduce enteric emissions while maintaining or increasing animal productivity (Arndt et al., 2022). Recently, it has become popular to measure large-scale exhaled metabolic gases such as methane (CH_4_) and carbon dioxide (CO_2_) emissions from cattle using the GreenFeed system (C-Lock Inc., Rapid City, SD). GreenFeed is a portable chamber system that measures individual animal gas production in real time (Hammond et al., 2016). Animals voluntarily use the machine, receiving pelleted bait feed to encourage visitation. Each animal visit should last at least 2 min to ensure the system records a valid measurement (Hammond et al., 2016). The sheer volume of data generated by GreenFeed on a 24-h basis and across weeks can be overwhelming. Many users struggle to process this daily influx of information effectively, often leading to mistakes and inconsistencies. To address these challenges, we adopted an approach that prioritizes the reproducibility of analyses by following the principle of “doing as little as possible by hand and as much as possible with functions” (Wickham and Bryan, 2023, p. xvi Preface). Motivated by this, we developed the R (R Core Team, 2022) package *greenfeedr*. The package offers functions for downloading, processing, and reporting GreenFeed data and is freely available at the Comprehensive R Archive Network (CRAN; https://cran.r-project.org/web/packages/greenfeedr/). In this article, we describe all functions implemented in the *greenfeedr* version 1.0.2 (Figure 1) and present examples based on real dairy cow data. All examples and code discussed in this article are available on GitHub at https://github.com/GMBog/greenfeedr.

**Figure 1:**
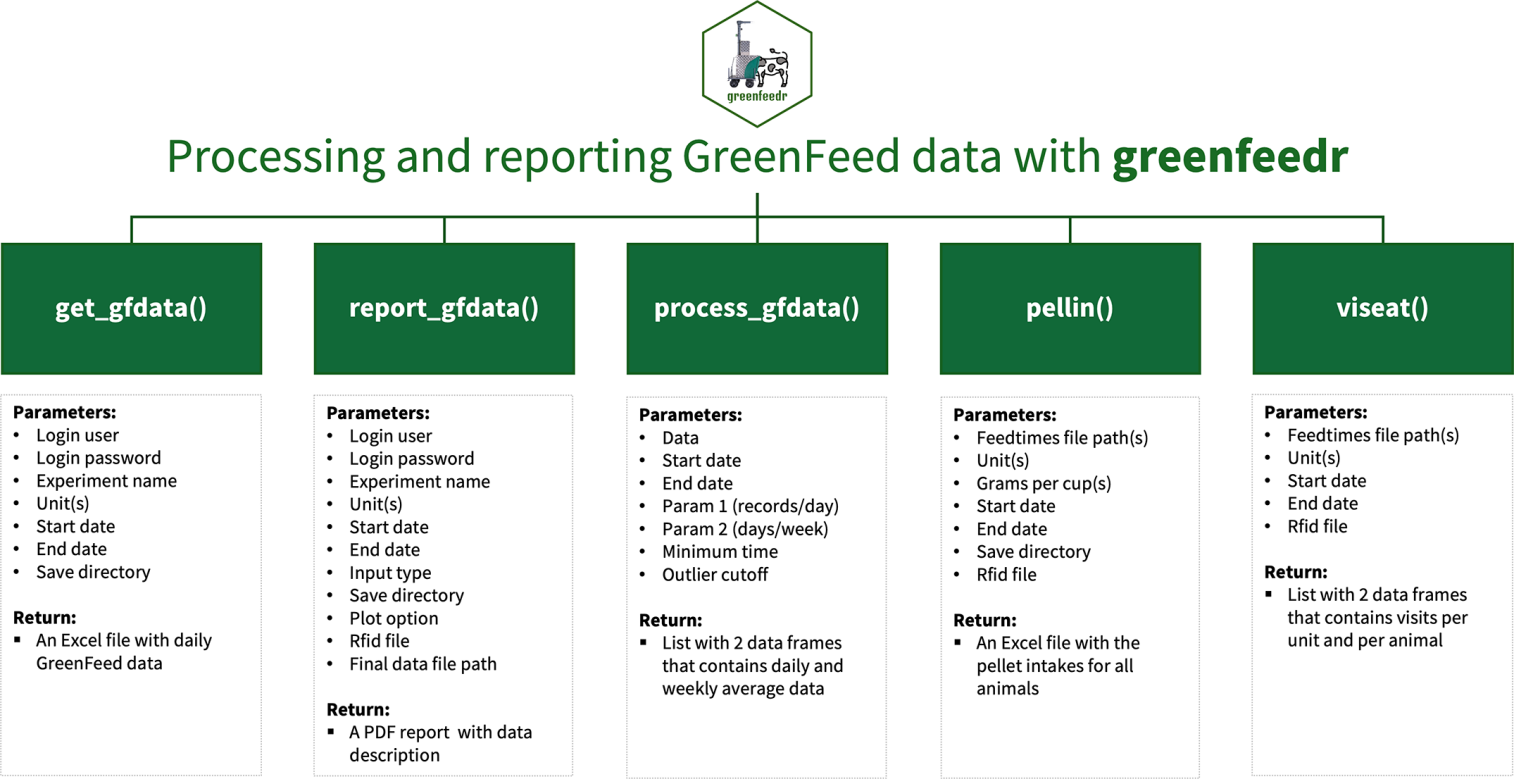
Components of R package *greenfeedr*.

For all GreenFeed users, the first step is to retrieve daily visit records (a record is generated after the visit has gone through the preprocessing system and converted into a usable data entry) from the C-Lock Inc. web interface. This seemingly simple task becomes burdensome when users simultaneously have many studies with multiple GreenFeed units. To address this problem, we developed a function named *get_gfdata*, which downloads daily visit records for a given time period for specific units requested by the user through an application programming interface (**API**) that interacts with the C-Lock Inc. server. Note that the API code is also available in the function *report_gfdata*, which generates daily reports from the user's data using R markdown. We developed the function *report_gfdata* to provide users with an easy-to-read report to check animals' visitation patterns and confirm that the GreenFeed is functioning correctly at the farm. If option ‘input_type = daily,' the function *report_gfdata* generates a ‘GreenFeed Daily Report' including (1) the number of animals using GreenFeed, including a list of specific animals (if users provide an animal list); (2) number of daily visit records per day, including the number of records with CH_4_ and CO_2_ production data; and (3) number of daily visit records per animal, including the distribution of records throughout the day using 4 time windows (i.e., 10 p.m. to 4 a.m., 4 to 10 a.m., 10 a.m. to 4 p.m., and 4 to 10 p.m., which are aligned with the typical behavior and physiology of ruminants), and the distribution of CH_4_ and CO_2_ production data per animal. Users can include one or more gases (by plot_opt) in their reports (i.e., CH_4_, CO_2_, O_2_, H_2_, or “all”). However, if option ‘input_type = final,' the function *report_gfdata* performs data processing using the finalized data provided by C-Lock Inc. to the users, typically 3 to 4 wk after the trial ends. Note that the ‘GreenFeed Final Report' created includes a plot of the variability of gas production across the day using all records without any filtering.

The main challenge is processing the high-dimensional data provided by GreenFeed for statistical analysis. For this purpose, we developed the function *process_gfdata*, which processes daily and finalized GreenFeed data. It looks similar to *report_gfdata*, but the main difference is that *process_gfdata* generates daily and weekly averages of gas production per animal and returns a list with 2 data frames, namely ‘daily_data' and ‘weekly_data.' Users can control data processing using 3 parameters: ‘param1' is the number of visit records per day to include in the daily average, ‘param2' is the number of days per week to include in the weekly average, and ‘min_time' is the minimum duration of an animal's visit records to be included for analysis (by definition, min_time should not be less than 2 min). The *greenfeedr* R package also contains 2 more functions, *pellin* and *viseat*, that use the ‘feedtimes' file provided by C-Lock Inc. The feedtimes file can be downloaded from the web interface and contains each animal visit, the number of food drops per visit, and the total number of visits in a day. Most GreenFeed users are interested in calculating daily pellet intakes from their experiments, so the function *pellin* returns an Excel (Microsoft Corp.) file with daily intakes per animal from all units used. Furthermore, the function *viseat* is helpful at the beginning of an experiment to check which animals are not visiting the GreenFeed unit(s), enabling the user to take quick actions to encourage animals to use the system. Note that a visit refers to when an animal enters its head into the unit, receives food drops, and exits the unit.

We illustrate the use of *greenfeedr* with an example using dairy cow data. The GreenFeed data came from a study with 32 mid-lactation Holstein cows conducted between January and March 2024 at the University of Wisconsin–Madison. The experimental protocol was approved by the Animal Care and Use Committee (IACUC #A006747). The data consisted of daily CH_4_ and CO_2_ (in g/d) visit records for 46 consecutive days using one GreenFeed unit in a freestall system. We defined 2 subsets of data: one with daily visit records (n = 2,298) until wk 4 (28 d) and another with daily visit records (n = 3,420) in the finalized data provided by C-Lock Inc. for all 7 wk (46 d). Furthermore, we explored the results obtained with the function *process_gfdata* by changing the parameters for param1 (1 to 3), param2 (3 to 7), and min_time (2 to 3; Table 1).

**Table 1:** Number of records and cows used to calculate daily and weekly methane (CH_4_) averages (mean ± SD) when different parameters of the function *process_gfdata* are defined^1^

param1	param2	Daily records	N cows	Daily CH_4_ (g/d)	Weekly records	N cows	Weekly CH_4_ (g/d)
min_time = 2							
1	3	1,257	32	376 ± 92	211	32	375 ± 64
	4				199	32	378 ± 62
	5				168	32	380 ± 61
	6				141	32	379 ± 61
	7				97	32	379 ± 60
2	3	938	32	373 ± 85	176	32	375 ± 63
	4				142	32	373 ± 62
	5				105	31	376 ± 62
	6				70	29	380 ± 66
	7				34	21	366 ± 56
3	3	596	32	377 ± 82	113	31	377 ± 67
	4				75	26	372 ± 66
	5				43	24	376 ± 71
	6				24	17	374 ± 75
	7				7	7	369 ± 91
min_time = 3							
1	3	1,113	32	378 ± 97	198	32	378 ± 65
	4				176	32	380 ± 64
	5				147	32	379 ± 63
	6				106	32	381 ± 66
	7				54	25	373 ± 62
2	3	674	32	376 ± 86	129	32	374 ± 68
	4				88	29	375 ± 67
	5				55	25	374 ± 68
	6				29	17	365 ± 55
	7				9	8	384 ± 65
3	3	363	32	376 ± 83	58	27	371 ± 65
	4				32	18	375 ± 74
	5				15	11	389 ± 57
	6				6	5	403 ± 77
	7				0	0	NA

1 param1 is number of records per day; param2 is the number of days with records; min_time is the minimum duration in minutes of a record. NA = not applicable.

Remarkably, all 32 cows were using the GreenFeed by d 28. Some cows were frequent users of the GreenFeed and provided many records (e.g., cow 24 with 184 visit records; 184/46 = 4 records per day). Other cows were less frequent users but still provided a decent number of records (e.g., cow 3 with 69 visit records; 69/46 = 1.5 records per day). Using the *report_gfdata* function, we determined that cows used the GreenFeed from early morning to late afternoon, and that gas records were well distributed throughout the day. The ‘final daily report' in Figure 2 shows the GHG variability across the day, demonstrating that CH_4_ is low early in the morning and increases 2 h after feeding (i.e., at ∼7 a.m.). Note that cows produced, on average, 375 ± 118 g/d (mean ± SD, CV 31%) of CH_4_ and 12,264 ± 1,967 g/d (mean ± SD, CV 16%) of CO_2_, and we observed a large range for both GHG (CH_4_ range: 67 to 809 g/d; CO_2_ range: 6,465 to 18,901 g/d).

**Figure 2:**
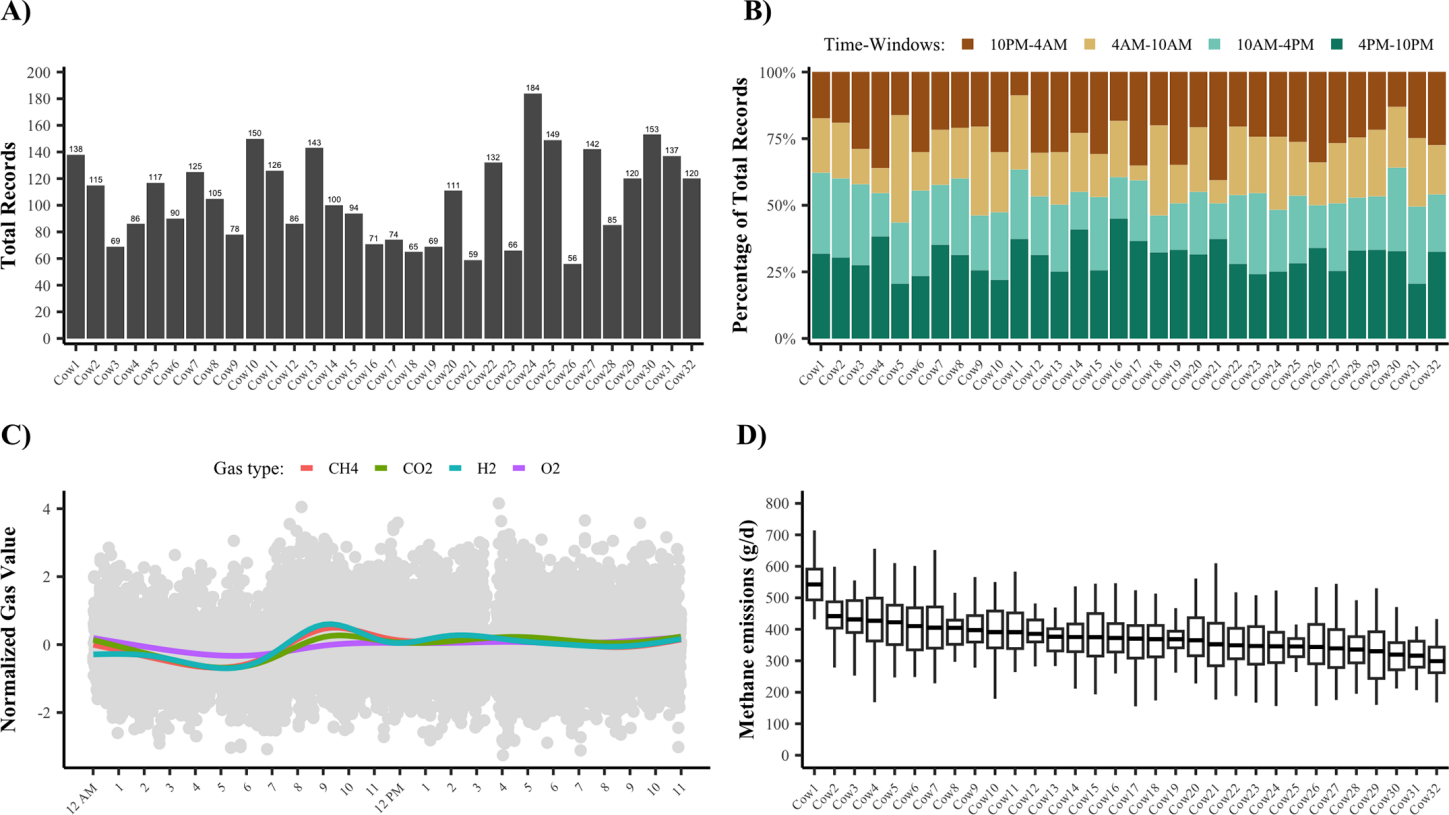
Description of GreenFeed data using *report_gfdata* function. (A) Number of records per animal. (B) Distribution of total records throughout 4 time windows. (C) Gas production throughout the day. Color lines represent the smoothed trend of each gas, and gray points are all visit records. (D) Distribution of methane emissions (g/d) per visit record and animal. The midline represents the mean, the box edges denote the first and third quartiles, and the whiskers extend to data points within 1.5 times the interquartile range.

We processed the 3,420 visit records (7 wk) using *process_gfdata*. The first step was to remove outliers for GHG production (mean ± 3·SD) using a minimum duration threshold defined as min_time = 2 min. We retained 99% of the initial data (3,408 visit records) as expected. Next, we computed weighted daily averages of GHG based on the visit record duration, including cows with at least 2 records per day (param1 = 2), and then weighted weekly averages based on the total visit record minutes, including cows with at least 4 d with records per week (param2 = 4). Cows in this study had on average 3 visit records per day, 18 visit records per week, and 5 d with visit records per week. These metrics show a good distribution of records across the study, which is crucial for the reliability of GHG measurements from the GreenFeed system. We obtained an average CH_4_ production of 373 ± 62 g/d (mean ± SD, CV 16.6%) and CO_2_ production of 12,241 ± 1,190 g/d (mean ± SD, CV 9.7%). Conveniently, the *process_gfdata* function gives users the ability to change the filtering parameters and define the best approach for their analysis. Table 1 shows the number of visit records and cows retained to calculate daily and weekly averages using different combination(s) of param1 (1 to 3 records), param2 (3 to 7 days with records), and min_time (2 and 3 min). The correlations suggest that both param1 and param2 influence the number of visit records and animals retained for analysis. Specifically, param1 is negatively correlated with the number of daily (−0.59 and −0.40) and weekly (−0.45 and −0.38) records and animals, whereas param2 shows a negative correlation with weekly (−0.37 and −0.26) records and animals. However, the correlation with methane emissions is less clear. For daily CH_4_, param1 shows a slight negative correlation (−0.28), whereas for weekly CH_4_, both parameters show weak positive correlations (0.10 for param1 and 0.01 for param2).

We described the capabilities of the *greenfeedr* to process and report GreenFeed data. Some functions, such as *report_gfdata* and *viseat*, can be helpful for daily checking of GreenFeed units at the farm, whereas others, such as *process_gfdata*, contribute to the proper description and evaluation of the experimental data. Note that this R package was written to be used with all species and for all systems (e.g., freestall, tiestall, and pasture). Overall, the *greenfeedr* package represents an important advancement in the management and analysis of GreenFeed data, offering a streamlined and efficient tool tailored to the needs of the user. By automating routine processes and providing robust data handling capabilities, this R package enhances reproducibility and saves time. Our illustration of the *greenfeedr* functions with dairy cow data demonstrates its effectiveness in simplifying complex workflows and generating insightful outputs that can inform management decisions.
